# Changes in life satisfaction over six years in the general population: A longitudinal study with the Satisfaction With Life Scale (SWLS)

**DOI:** 10.1371/journal.pone.0316990

**Published:** 2025-01-13

**Authors:** Andreas Hinz, Anja Mehnert-Theuerkauf, Heide Glaesmer, Matthias L. Schroeter, Ana N. Tibubos, Katja Petrowski, Michael Friedrich

**Affiliations:** 1 Department of Medical Psychology and Medical Sociology, University of Leipzig, Leipzig, Germany; 2 Max Planck Institute for Human Cognitive and Brain Sciences, Leipzig, Germany; 3 Clinic for Cognitive Neurology, University Hospital Leipzig, Leipzig, Germany; 4 Department of Diagnostics in Healthcare and E-Health, University of Trier, Trier, Germany; 5 Department of Psychosomatic Medicine and Psychotherapy, University Medical Center of the Johannes Gutenberg University Mainz, Mainz, Germany; 6 Department of Medical Psychology and Medical Sociology, University Medical Center of the Johannes Gutenberg University Mainz, Mainz, Germany; University of Jyvaskyla, FINLAND

## Abstract

**Background:**

Satisfaction with life is a key concept for most individuals. The Satisfaction With Life Scale (SWLS) for measuring general life satisfaction has been widely analyzed in terms of cross-sectional associations. However, the knowledge about long-term changes in life satisfaction and the associations between such changes and changes in other variables of physical and mental health is limited.

**Methods:**

A community-based representative sample of the general population has been examined twice with a time interval of six years (n = 4,999), using the SWLS and several other scales.

**Results:**

Over the six years, the mean SWLS score of the total sample remained nearly unchanged (M = 27.0, SD = 5.2, both at t1 and at t2). The test-retest correlation was *r*_tt_ = 0.66 for the total sample, and there were only marginal differences in temporal stability between male and female respondents. Changes in the SWLS over the six years were correlated with changes in optimism (*r* = 0.23), mental health (*r* = 0.26), social functioning (*r* = 0.22), perceived social support (*r* = 0.21), anxiety (*r* = -0.30), and physical complaints (*r* = -0.18). These change score correlations were lower than the corresponding coefficients under the cross-sectional perspective. Measurement invariance across sex, age, and time was established.

**Conclusion:**

The SWLS proved to be an appropriate tool for assessing changes in life satisfaction, and correlations between change scores of life satisfaction and health-related variables complement the knowledge about these associations from a cross-sectional perspective.

## Introduction

Life satisfaction is the cognitive evaluation of subjective well-being. It is a goal shared by most individuals, and results of life satisfaction research can be used by policy makers and stakeholders [1,2]. Sometimes, the terms life satisfaction and subjective well-being are regarded as interchangeable [3]. In recent decades, research on life satisfaction has increasingly gained importance [4]. Life satisfaction is correlated with personality traits [5,6], depression [7], flexible goal adjustment [8], and perceived social support [9]. For clinicians, the concept of life satisfaction is of special interest, since life satisfaction is associated with mental and physical health [10,11], quality of life [6], several specific disorders and chronic diseases [11], specific symptoms such as incontinence [12], illness acceptance and stigmatization [13], need of informal care [14], and even mortality [15].

The Satisfaction With Life Scale SWLS [16] is a frequently used instrument for assessing life satisfaction. Multiple studies have been performed to test the psychometric quality of the scale. Most studies found that the one-dimensional structure yields good fit indices, but that a modified one-factor model allowing for correlated errors between items 4 and 5 performed even somewhat better [17,18]. Cronbach’s α coefficient generally reaches scores above 0.80 [8,19,20] or even above 0.90 [17,18]. Measurement invariance of the scale concerning age and sex has been established in several studies [4,20,21]. The SWLS has been applied to large and representative samples of the general population in several countries, e.g., Norway [17], Germany [22], Denmark [10], Spain [11], Mexico [23], and South Korea [24].

Despite the large body of knowledge on the factors affecting life satisfaction and the psychometric properties of the SWLS, the knowledge about long-term changes in life satisfaction and the factors accounting for such changes is limited.

Test-retest correlations of the SWLS have been studied for time intervals of several weeks [16,19,25], but studies on long-term changes are rare. In particular, it has not been systematically tested yet whether there are systematic age and sex differences in the temporal stability of life satisfaction, and to what degree changes in life satisfaction are associated with changes in other related constructs of mental health. Regarding measurement invariance, several studies tested measurement invariance across age and sex in cross-sectional studies [4,18]; however, the measurement invariance across time points, based on a large general population study, is missing until now. Multiple psychometric tests of the SWLS have been performed on the basis of cross-sectional studies. A longitudinal study, however, is necessary to test the psychometric properties of the items and the scale when considering the measurement of changes in life satisfaction, such as discrimination power of the items regarding change scores from baseline to follow-up measurement. For these reasons, we performed a longitudinal study with a large sample of the general population to address these issues.

In 2017, we initially published results of a cross-sectional study on life satisfaction with 10,000 participants from the general population [26]. Six years after this first assessment, the participants were invited to take part in a follow-up study. Based on the data of this follow-up study, the objectives of this paper were (a) to analyze changes in life satisfaction in a six-year time period, (b) to test psychometric properties of the SWLS including temporal stability, reliability of change, and measurement invariance across time, and (c) to examine the associations between life satisfaction and other variables (anxiety, social support, habitual optimism, bodily complaints, and quality of life) both from a cross-sectional and from a longitudinal perspective.

## Material and methods

### Sample

The LIFE-Adult-Study, conducted by the Leipzig Center for Civilization Diseases (LIFE), is a population-based study with a representative sample of people living in the city of Leipzig, Germany. Between August 1^st^ 2011 and November 30^th^ 2014, the first wave of this study (n = 10,000) was conducted. From the local residents’ registration office, we obtained an age- and gender- stratified random selection of inhabitants, ranging in age from 18 to 80 years, with the focus on the age group 40–80 years. At the study center, the participants underwent several assessment batteries: several medical examinations, medical history, lifestyle factors, and a set of questionnaires regarding physical, mental and social factors, with an assessment duration of about six hours. Details of the study design [27] and basic results of the study [28] have been published elsewhere. Written informed consent was obtained from all participants.

About six years after the t1 assessment, all reachable participants of the baseline examination were invited to attend a follow-up assessment (t2). This follow-up was conducted from October 1^st^ 2017 to August 31^st^ 2021. Those participants who were willing and able to participate in the follow-up examination were sent a letter with multiple questions and questionnaires (including the SWLS) by mail. Analyses of the SWLS based on the t1 assessment have already been published [26]. The present paper adds the results of the longitudinal analyses. The study was approved by the ethics committee of the Medical Faculty of the University of Leipzig, approval number 201/17-ek.

### Instruments

The SWLS [16] is a five-item instrument for measuring satisfaction with life. The scale comprises five statements (e.g., “I am satisfied with my life”) with seven response options ranging from 1 (strongly disagree) to 7 (strongly agree). This results in a sum score range from 5 to 35.

The following instruments were also included both at baseline and at follow-up: the Generalized Anxiety Disorder Screener GAD-7 [29] for measuring anxiety, the Short Form Health Survey–8 SF-8 [30] for measuring quality of life, the Patient Health Questionnaire PHQ-15 [31] for measuring bodily complaints, the Life Orientation Test-Revised LOT-R [32] for measuring dispositional optimism, and the ENRICHD Social Support Scale [33] for measuring social support. Sociodemographic and behavioral factors were obtained in a structured interview.

### Statistical analysis

Mean score differences between two groups of participants (e.g., men vs. women) were expressed with effect sizes (Cohen’s *d*) and tested with *t*-tests for independent samples. Cronbach’s α coefficient was used to quantify internal consistency. Coefficients of temporal stability were calculated with Pearson’s correlation coefficients. Though some researchers prefer to use intra-class-coefficients, most of the research on temporal stability in the literature uses Pearson correlation coefficients, and for reasons of comparability with these studies we also used these more common correlation coefficients.

To test psychometric properties of the single items, we used discriminatory power coefficients. In addition, we calculated discriminatory power analyses with the change scores. These coefficients express to what degree the change from t1 to t2 in a single item corresponds with the change of the scale after removing the item of interest. These analyses were performed with IBM SPSS Statistics version 27.

Analyses of measurement invariance were performed using R, Version 4.1.1 [34]. Model evaluations in R used the packages lavaan 0.6.9 [35] and semTools 0.5.5 [36]. All models were estimated using the diagonally weighted least squares (DWLS) method with mean- and variance-adjusted test statistic. Model fit was judged using a combinational rule of comparative fit index (CFI) and standardized root mean square residual (SRMR) [37]. Based on this rule, poor fit was indicated if both CFI and SRMR exceed their threshold for acceptability, i.e., CFI < 0.95 and SRMR > 0.06. In addition, we calculated the Tucker-Lewis Index (TLI) and the root mean square error of approximation (RMSEA). Differences in model fit were evaluated by the difference of CFI values (ΔCFI) between sequential models. A difference of at least 0.002 or above, respective -0.002 or below, was regarded as a substantial increase, respective decrease in model fit [38]. At the beginning, we tested the models at each occasion (t1 and t2) separately. An unconstrained model in which both occasions were combined served as the baseline model. Acceptable fit of this model shows configural invariance, that is, the factor patterns remain stable over time (configural invariance). This model was the starting point for detecting any violation of measurement invariance. The procedure followed a forward approach of releasing parameter constraints, i.e., parameters were constrained set by set, and released, if necessary, in the following order: thresholds and weights (metric or weak invariance), then additionally intercepts (scalar or strong invariance), and finally residuals (full or strict invariance).

## Results

### Sample description

The baseline assessment included 10,000 individuals, 9,711 of which completed the SWLS at t1. The response rate of this baseline assessment was 33% since initially about 30.000 individuals were invited to participate. All available participants of the t1 assessment were asked to take part in the follow-up study, and 4,999 agreed to do so and provided valid SWLS data both for the t1 and the t2 assessment. Characteristics of these participants are displayed in Table 1.

**Table 1:** Sociodemographic characteristics of the sample at t1 (n = 4,999).

	n	%
Sex		
Male	2,358	47.2
Female	2,641	52.8
Age		
Mean, SD (years)	M = 57.1	SD = 12.3
Age group		
≤ 39 years	270	5.4
40–49 years	1,265	25.3
50–59 years	1,127	22.5
60–69 years	1,358	27.2
≥ 70 years	979	19.6
Marital status ^(^^a^^)^		
Married, living together	3,181	63.7
Married, living separately	101	2.0
Never married	832	16.6
Divorced	611	12.2
Widowed	272	5.4
Education ^(^^a^^)^		
< 10 years	331	6.7
10–11 years	2,680	54.1
≥ 12 years	1,939	39.2
Occupational status ^(^^a^^)^		
Working full time	2,252	45.4
Working part-time	465	9.4
Unemployed	222	4.5
Retired	1,914	38.6
Other	108	2.2
Socio-economic status ^(^^a^^)^		
Low	755	15.1
Medium	3,051	61.1
High	1,186	23.8

^(a)^ Missing data not reported.

### SWLS mean scores and item characteristics

Table 2 presents item and scale mean scores, effect sizes for the differences between the t2 and t1 assessments, and coefficients of discriminative power. The t1 and t2 SWLS mean scores were nearly identical, with a negligible difference of 0.05 units. All items contributed positively to the total score with discrimination power coefficients between 0.63 and 0.79. Cronbach’s α was good with α = 0.88 at t1 and α = 0.89 at t2.

**Table 2:** SWLS item and sum score characteristics.

Item	t1		t2		Diff. t2-t1		ES t2-t1	discr. power t1	discr. power t2	discr. power (Δitem, Δscale)	*r* (t1, t2)
	M	SD	M	SD	M	SD					
I 1	5.31	1.16	5.25	1.20	-0.06	1.15	-0.05	.77	.79	.57	.52
I 2	5.46	1.17	5.47	1.19	0.01	1.14	0.01	.74	.74	.56	.54
I 3	5.70	1.09	5.65	1.12	-0.05	1.08	-0.05	.78	.79	.62	.52
I 4	5.63	1.14	5.62	1.15	-0.01	1.10	-0.01	.76	.77	.56	.54
I 5	4.90	1.62	4.97	1.16	0.06	1.49	0.05	.63	.64	.39	.56
Sum score	27.01	5.17	26.96	5.24	-0.05	4.29	-0.01	α = .88	α = .89	α = .76	.66

Note: ES: Effect size of the difference t2 score—t1 score; discr. power at t1; discriminative power; discr. power (Δitem, Δscale): Discriminative power of the items regarding the change from t1 to t2; *r* (t1, t2): Test-retest correlation.

Concerning the change scores (difference between the t2 and the t1 score), the α coefficient was 0.76, and the coefficients of discrimination power of these item change scores were about 0.20 lower than the corresponding coefficients of the raw scores. Item 5 showed the weakest contribution to the total score in all analyses: t1, t2, and change.

The SWLS mean score of those participants of the t1 examination who did not take part in the follow-up (drop out; n = 4,812) was lower (25.9 ± 5.87) than the mean score of those who completed t2 as well (27.0 ± 5.17). The mean age of this subgroup of drop-outs was 56.3 years, and 52.1% were female, compared with a mean age of 57.1 years and a proportion of women of 52.8% in the group of the complete respondents. The effect size of the SWLS score at t1 between the completers and the drop-outs was *d* = 0.20.

### Age and sex differences in the course of satisfaction with life

Table 3 presents the SWLS mean scores and change scores for age and sex groups separately. While in the total sample there was no mean score change, the youngest (18–39 years) and the oldest (70 years and above) age group showed a certain decline in satisfaction with life.

**Table 3:** Mean scores, effect sizes, and test-retest correlations for the SWLS core.

	N	t1		t2		Diff. (t2-t1)		*d*	*p*	*r*_tt_
		M	(SD)	M	(SD)	M	(SD)			
Men										
18–39 years	137	27.1	(4.43)	26.5	(5.24)	-0.55	(4.70)	-.11	.175	.54
40–49 years	551	26.5	(5.50)	26.8	(5.54)	0.30	(4.11)	.05	.090	.72
50–59 years	506	25.9	(5.45)	26.5	(5.33)	0.58	(4.48)	.11	.004	.65
60–69 years	661	27.3	(5.03)	27.7	(4.77)	0.40	(4.15)	.08	.013	.64
≥ 70 years	503	28.0	(4.12)	27.2	(4.72)	-0.82	(3.88)	-.19	< .001	.62
All men	2,358	27.0	(5.08)	27.0	(5.12)	0.09	(4.22)	.02	.254	.66
Women										
18–39 years	133	27.2	(5.39)	26.8	(6.36)	-0.41	(5.54)	-.07	.400	.57
40–49 years	714	26.9	(5.23)	26.8	(5.36)	-0.15	(4.20)	-.03	.340	.69
50–59 years	621	27.1	(5.34)	27.2	(5.33)	0.07	(4.28)	.01	.673	.68
60–69 years	697	26.9	(5.26)	26.8	(5.20)	-0.04	(4.30)	-.01	.785	.66
≥ 70 years	476	27.4	(5.00)	26.7	(5.24)	-0.69	(4.33)	-.13	< .001	.64
All women	2,641	27.1	(5.25)	26.9	(5.34)	-0.18	(4.35)	-.03	.034	.66
Total	4,999	27.0	(5.17)	27.0	(5.24)	-0.05	(4.29)	-.01	.429	.66

The temporal stability (*r*_tt_) was between 0.54 and 0.72, with the lowest stability (*r*_tt_ < 0.60) in the youngest age group. The stability of the SWLS score in the oldest age group was only marginally lower (0.62 and 0.64 for men and women, respectively) than the stability of the total groups of men and women (0.66 for both sexes).

### Measurement invariance

The results of the measurement invariance analyses are presented in Table 4. The (unidimensional) model of the SWLS showed acceptable fit since the conditions of CFI ≥ 0.95 and SRMR ≤ 0.06 were fulfilled at each occasion and in the combination of both occasions (configural invariance). After restricting thresholds and weights to equality in both occasions, the model fit increased from CFI = 0.992 (configural invariance) to CFI = 0.994 (metric invariance) by a value of ΔCFI = 0.0025. Restricting intercepts (scalar invariance) and residuals (full invariance) did also not lead to a substantial decrease in model fit (ΔCFI < 0.002). In the full invariance model (latent mean at t1 fixed to 0, variance to 1), the latent mean at t2 changed by -0.01 standard deviations. This change can be considered negligible.

**Table 4:** Testing for measurement invariance across t1 and t2.

	NPar	Chi^2^ (df) p-value	Chi^2^/df	CFI	SRMR	TLI	RMSEA	ΔCFI	ΔSRMR	ΔTLI	ΔRMSEA
**t1**	35	490.8 (5) <0.001	98.2	0.992	0.025	0.985	0.139	.	.	.	.
**t2**	35	484.9 (5) <0.001	97.0	0.993	0.024	0.986	0.139	.	.	.	.
**t1 and t2**	76	915.4 (29) <0.001	31.6	0.992	0.023	0.987	0.078	.	.	.	.
**Configural invariance**	76	915.4 (29) <0.001	31.6	0.992	0.023	0.987	0.078	.	.	.	.
**Metric invariance**	52	664.3 (53) <0.001	12.5	0.994	0.024	0.995	0.048	0.0025	0.0006	0.0078	-0.0302
**Scalar invariance**	48	701.1 (57) <0.001	12.3	0.994	0.024	0.995	0.048	<0.002	<0.0001	0.0001	-0.0005
**Full invariance**	43	733.8 (62) <0.001	11.8	0.994	0.024	0.996	0.047	<0.002	<0.0001	0.0002	-0.0010

**Note.** NPar: Number of parameters; Chi^2^: Scaled chi-squared statistic; df: Degrees of freedom; p-value: Type-I error probability; CFI: Scaled comparative fit index, SRMR: Standardized root mean square residual; TLI: Scaled Tucker-Lewis index; RMSEA: Scaled root mean square error of approximation; Δ indicates the difference of fit indices between sequential (nested) models.

### Correlations between the SWLS and other scales

Table 5 presents the correlations between the SWLS scores and several other scales both from a cross-sectional and a longitudinal perspective. The highest cross-sectional correlations (coefficients above 0.40) were found for anxiety (inverted), social support, optimism, mental health, and the Mental Component Summary (MCS) score of the SF-8. The right column of Table 5 shows the correlations between changes in life satisfaction and changes in the other variables. The coefficients were weaker than the cross-sectional correlations, but the sequence of the correlations was similar to that of the cross-sectional coefficients, with highest coefficients for anxiety (inverted) and the MCS score of the SF-8. This means that participants who become less anxious during the six-year period or who improved their mental health also gained in life satisfaction during that time period.

**Table 5:** Correlations between the SWLS and several other scales.

	r (SWLS, scale) at t1	r (SWLS, scale) at t2	r (Δ SWLS, Δ scale)
GAD-7: Anxiety	-.41	-.46	-.30
ENRICHD: Social Support	.41	.44	.21
LOT-R Optimism	.44	.49	.23
LOT-R Pessimism	-.34	-.40	-.21
PHQ-15: Complaints	-.32	-.39	-.18
SF-8: Physical functioning	.24	.28	.13
SF-8: Role-physical	.29	.30	.13
SF-8: Bodily pain	.22	.26	.09
SF-8: General health	.37	.38	.17
SF-8: Vitality	.39	.37	.21
SF-8: Social functioning	.37	.39	.22
SF-8: Role-emotional	.36	.37	.21
SF-8: Mental health	.41	.42	.26
SF-8: Physical Component Summary	.26	.28	.11
SF-8: Mental Component Summary	.43	.44	.28

Note. All correlations are statistically significant at p<0.001.

## Discussion

The main aim of this study was to investigate changes in life satisfaction over a period of six years. The SWLS mean score remained nearly unchanged (M = 27.0 at both time points), though the participants became six years older. This lack of mean score change was also observed in other studies [39,40].

The test-retest correlation between the two measurements of the SWLS was *r*_tt_ = 0.66, which means that 44% of the variance of the t2 assessment can be explained by the scores obtained six years before. This correlation is lower than the coefficients reported from other studies, however, the time intervals were shorter in these studies: *r*_tt_ = 0.82 [16] (2 weeks), *r*_tt_ = 0.74 (15 days) [25], *r*_tt_ = 0.81 [21] (four weeks), *r*_tt_ = 0.70 [19] (10 weeks), and ICC = 0.73 [40] (one year). A longitudinal study with up to 29 years between t1 and t2 reported a correlation of only *r*_tt_ = 0.25 between the measurement points. However, in that study the SWLS was only used at t2, while at t1 life satisfaction was assessed with a single question [6]. Therefore, it cannot be inferred to what degree the long temporal distance and the different assessment tools contributed to this low coefficient. The results underline that the temporal stability depends on the time interval between the measurement points, and that longer time intervals understandably lead to lower temporal stability coefficients. One large general population study using the SWLS with time intervals of three years has been published [39]; however, this study did not report coefficients of temporal stability.

The sample size of our study was large enough to calculate coefficients of temporal stability separately for sex and age groups. It is interesting to note that the oldest and the youngest age groups showed the lowest stability coefficients, and that the stability was highest in the age range 50–69 years. Possible explanations are that in the youngest group there are more changes in the objective life situation and the corresponding challenges (e.g., changes in the marital and professional situation), and in the oldest age group changes may occur with respect to losing a spouse or experiencing severe health problems. However, the sample size of the youngest age group was relatively low, and the difference between the stability scores of the oldest age group and the other groups were also small in magnitude, which limits the generalizability of these findings. Further research on the stability of life satisfaction is therefore needed, in particular in persons who experience severe diseases.

The psychometric quality and the cross-sectional measurement invariance of the SWLS in general population samples have already been established in multiple cross-sectional studies [4]. The results of our study complement these findings by also proving the measurement invariance over time, which supports the findings from studies with Turkish [21] and Serbian [41] university students.

Regarding the contribution of the five items to the overall score of the SWLS, we first confirmed previous findings that all items positively contributed to the total scale and that the contributions of items 4 and 5 were somewhat lower than those of items 1 to 3 [18,20]. This pattern could also be shown from the longitudinal perspective. Reliability of change quantifies to what degree the items are uniform not in constructing the (cross-sectional) total score but the total change score. All change score *r*_it_ coefficients were positive and somewhat lower than the corresponding *r*_it_ coefficients from the cross-sectional calculations. Moreover, the two items with the lowest cross-sectional *r*_it_ scores (items 4 and 5) also showed the lowest reliability of change scores.

The cross-sectional correlations between the SWLS and other constructs regarding mental and physical health confirmed the expected relationships, with higher associations with scales of mental health in comparison with those of physical health. Regarding the change scores, the correlations were weaker, but nevertheless existent, with a similar sequence as those of the cross-sectional associations. Until now, there are only few applications of such change score correlations, e.g., [39,42,43], and such coefficients complement the knowledge about the correlational structure of scales.

An accidental finding was that most correlations between the SWLS scores and related relevant constructs (Table 5) were somewhat higher at t2 in comparison to the t1 measurement. Among the 15 comparisons, there was only one variable (SF-8: vitality) with a higher correlation at t1. One reason for the higher correlations at t2 may be that the participants filled in the questionnaire at their homes at t2. This might have contributed to a more consistent way of responding to the questionnaires. Cronbach’s α, however, which is also an indicator for the consistency in responding, was only marginally higher at t2 (α = 0.89) in comparison to t1 (α = 0.88), and no difference between the 14 pairs of correlations in Table 5 exceeded the value of 0.05. Therefore, we cannot generalize the finding of higher correlations in a follow-up measurement compared to baseline measurements. However, the question whether the waves of a longitudinal study can always be considered as comparable with regard to the consistency of the respondents is a matter for future research.

Attempts have been made to assess life satisfaction with a one-item instrument [44,45] and to compare the results with those of the 5-item SWLS. The correlations between that one-item scale and the SWLS were 0.73 and 0.67. Our results show that the temporal stability coefficients of all single items were lower than the stability coefficient of the total scale (*r* = 0.66), and that the corrected test-item correlations (coefficients of discriminative power) were markedly lower than the α coefficients at both time points. This argues in favor of using the 5-item scale for the sake of higher reliability and not switching to a one-item instrument.

The SWLS was designed for measuring general life satisfaction. That broad concept consists of satisfaction components regarding multiple dimensions, e.g., physical and mental health, finances, occupation, and social relationships including partnership. These components are more or less correlated with general life satisfaction. Moreover, these specific components of satisfaction can be more or less correlated with objective scores. A general population study, for example, found that general life satisfaction as measured with the SWLS correlated more strongly with satisfaction with finances (*r* = 0.59) in comparison to satisfaction with health (*r* = 0.49), and that satisfaction with finances was more strongly correlated with the objective finance status (*r* = 0.36) than the correlation between satisfaction with health and objective health status (*r* = 0.26) [46]. The changes in general satisfaction that were analyzed in our study only refer to general satisfaction with life, and a separate analysis of such components of satisfaction is a matter for future research.

### Limitations

Only about half of the participants of the baseline assessment also took part in the t2 examination. The completers were more satisfied with life than the drop-outs, with a significant effect size of *d* = 0.20. Moreover, even the 10,000 participants of the t1 assessment were healthier than those who did not take part in the study at all [47]. This means that the total SWLS mean score obtained in our study may be affected by this bias. However, for the analyses regarding change, age and sex differences, and associations with other relevant constructs, we believe that this kind of bias does not substantially influence the results. While the t1 assessment was performed in the study center, the t2 assessment was done at home, which might also have contributed to differences in the assessments. Finally, the sample size of the age range 18–39 years was not large enough to derive generalizable conclusions.

## Conclusions

In summary, the SWLS proved to be a reliable instrument for assessing life satisfaction in longitudinal studies. Sex was not a significant predictor of life satisfaction or changes in life satisfaction. The correlations of the change scores proved to be useful tools for understanding the (longitudinal) relationship between life satisfaction and other relevant psychosocial or health-related variables.
